# Omitting Elective Pelvic Nodes Irradiation in High Risk Prostate Cancer: Report on 43 Consecutive Elderly Patients

**DOI:** 10.3390/jpm16040177

**Published:** 2026-03-24

**Authors:** Emanuele Chioccola, Mara Caroprese, Christina Amanda Goodyear, Angela Barillaro, Gianluca Valerio, Caterina Oliviero, Mauro Buono, Stefania Clemente, Antonio Farella, Manuel Conson, Roberto Pacelli

**Affiliations:** 1 University Hospital Federico II, 80131 Napoli, Italy; emanuele.chioccola@unina.it (E.C.); maracaroprese@gmail.com (M.C.); christina.goodyear3@gmail.com (C.A.G.); angela.barillaro@unina.it (A.B.); gianluca.valerio1294@gmail.com (G.V.); caterina.oliviero@gmail.com (C.O.); mauro.buono88@gmail.com (M.B.); stefaniaclementesc@gmail.com (S.C.); antonio.farella@unina.it (A.F.); manuel.conson@unina.it (M.C.); 2 Department of Advanced Biochemical Sciences, University Federico II, 80131 Napoli, Italy

**Keywords:** prostate cancer, elderly patients, radiotherapy, nodal irradiation

## Abstract

**Background**: Radiotherapy (RT) combined with androgen deprivation therapy (ADT) is a standard treatment for non-metastatic high-risk (HR) prostate cancer (PC). However, the benefit of elective nodal irradiation (ENI) in clinically node-negative (cN0) patients, although suggested, remains controversial, particularly in the elderly. We report the outcomes of elderly HR PC patients treated with prostate-only RT (PORT) and ADT in a “real-word” setting. **Methods**: Between 2016 and 2022, 43 consecutive elderly patients (median age 76 years) with HR- or very HR-PC according to NCCN criteria version 1.2026 (cN0, cT3-cT4 and/or ISUP Grade Group 4–5 and/or PSA serum levels at diagnosis ≥ 20 ng/mL) were treated at our institution. All patients were staged with abdominal MRI or CT and bone scan; nineteen patients (44.2%) also underwent 68Ga-PSMA-11 or 18F-fluorocholine PET/CT. All patients received PORT (predominantly moderate hypofractionation, 67.5–70 Gy in 25–28 fractions) and ADT (median duration 24 months). To ensure consistency, all oncological endpoints—Biochemical Failure-Free Survival (BFFS; Phoenix criteria), Disease-Free Survival (DFS), Metastasis-Free Survival (MFS), Prostate Cancer-Specific Survival (PCSS), and Overall Survival (OS)—were calculated from a unified time-zero (initiation of first oncological treatment). DFS was defined as a composite endpoint including biochemical failure, radiological recurrence, or initiation of salvage therapy. **Results**: at a median follow-up of 60 months, no patient reached the Phoenix threshold, resulting in a 100% 5- and 7-year BFFS. However, 4 patients (9.3%) experienced radiological recurrence detected via PET/CT before reaching the nadir + 2 threshold, yielding an estimated 5-year and 7-year DFS of 94.7% and 71.8%, respectively. The 5- and 7-year MFS was of 97.6% and 88.7%, respectively. Seven deaths occurred, all non-PC related, resulting in a 5-year OS of 86.7% and a Prostate Cancer-Specific Survival of 100%. Gastrointestinal toxicity was notably low (no acute or late G3-G4 events). **Conclusions**: Our findings suggest that PORT, when combined with long-term ADT and modern staging, provides excellent disease control and a favorable safety profile in elderly HR PC patients. Given the high rate of competing mortality in this population, treatment de-escalation via PORT appears to be a clinically reasonable strategy. These results are hypothesis-generating and warrant validation in prospective randomized trials.

## 1. Introduction

Prostate cancer (PC) is the second most frequent cancer worldwide and the most diagnosed tumor among men in over half of all countries, including Western Europe. International variations in incidence rates are primarily due to differences in diagnostic practices and screening protocols. According to the National Cancer Institute, the median age at diagnosis is 67 years [1].

Over the last few decades, the impact of risk factors—such as clinical T stage, biopsy Gleason Score (ISUP Grade Group), and pre-treatment PSA levels—on the prognosis of PC patients has been well-established [2,3]. This led to the development of several risk stratification models, starting with the landmark criteria published by D’Amico et al. in the 1998 [4,5,6]. It is estimated that 15% of newly diagnosed PCs exhibit high risk features [7]. Prognosis varies significantly according to the number of risk factors present at diagnosis; the presence of two or more factors is associated with a dramatic reduction in biochemical recurrence-free survival and increased overall mortality [8,9]. In high-risk (HR) PC patients, a median time to biochemical failure of 32.5 months has been observed after definitive radiotherapy (RT). Furthermore, an association has been established between this outcome and clinical recurrence, development of distant metastases and the PC-specific mortality [10,11,12].

The combination of RT and androgen deprivation therapy (ADT) is a standard-of-care for HR localized PC, demonstrating superiority over either modality alone [13,14] and yielding outcomes comparable to radical prostatectomy [15]. However, the optimal duration of ADT remains a subject of debate. While long-term therapy improves oncological outcomes, it can lead to significant metabolic and cardiovascular toxicities; thus, the duration must be tailored to the patient’s comorbidity profile [16,17].

With regard to RT modalities, the favorable impact of dose-escalation, along with the effectiveness and feasibility of non-conventional fractionation schedules (such as moderately hypofractionated RT or stereotactic ablative RT), has been established [18,19]. However, the decision to include pelvic lymph node irradiation for HR-PC patients with localized or locally advanced disease—but without clinical nodal involvement—remains controversial. Given the potential for increased toxicity, the clinical benefit of elective nodal irradiation (ENI) has yielded contrasting results in both retrospective and prospective studies, particularly in patients receiving concurrent long-term ADT [20,21,22,23].

For elderly PC patients, the presence of relevant comorbidities is the strongest predictor of overall survival (OS). Comorbidities influence not only survival but also patients’ compliance with oncological treatments and their tolerance to side effects, thereby impacting quality of life during and after therapy [24].

We report a single-institution experience treating a consecutive elderly HR-PC group of patients (median age 76 years; range 65–82) with prostate-only radiation therapy (PORT) combined with ADT, focusing on oncological control and toxicity in a “real-world” geriatric setting.

## 2. Materials and Methods

### 2.1. Population Selection

This study retrospectively evaluated a consecutive cohort of patients aged 65 or older with HR- or very-HR-PC, according to the 2026 NCCN Risk Group classification at diagnosis [25], treated at our Institution between August 2016 and June 2022. In line with previous geriatric oncology literature and clinical trials, the threshold of 65 years was adopted to define the elderly population [26]. All patients underwent definitive RT combined with ADT. Inclusion criteria were: histological diagnosis of prostatic adenocarcinoma; clinical staging of cT3-cT4 and/or ISUP Grade Group 4–5 [27] and/or baseline total PSA ≥ 20 ng/mL; no clinical or radiological evidence of nodal involvement (cN0) and distant metastasis (cM0) at staging; ADT (androgen suppression using LHRH analogs and/or antiandrogen steroid drugs) administrated for a minimum of 6 months; and completion of the prescribed definitive RT course.

All patients underwent staging with abdominal MRI and/or contrast-enhanced CT, as well as total-body bone scintigraphy and/or PET/CT scan (using 68Ga-PSMA-11 or 18F-fluorocholine). The risk of microscopic nodal involvement was retrospectively estimated using the Roach equation and the Memorial Sloan Kettering Cancer Center (MSKCC) nomogram [28,29,30]. At baseline, comorbidities and ongoing medical therapies were recorded; cardiovascular risk factors—including age, tobacco smoking, hypertension, hypercholesterolemia, and diabetes—were defined according to the “Progetto Cuore” guidelines of the Italian National Institute of Health (ISS) [31].

Patients who did not meet all the above criteria or had incomplete follow-up data were excluded to ensure a clinically homogeneous population. Further exclusion criteria included prior prostatectomy, previous pelvic RT and/or the presence of hip prostheses. During RT, patients underwent weekly medical evaluations. Follow-up was scheduled in accordance with ASCO Survivorship Care Guidelines [32]: every 3 months for the first 2 years, every 6 months until the fifth year, and annually thereafter. Clinical assessments included a complete physical examination, digital rectal examination (DRE), and PSA monitoring; acute and late toxicities were recorded. Treatment-related toxicities were reported following the grading proposed by the Radiation Therapy Oncology Group (RTOG) scale, categorized as acute if occurring during or within 3 months after RT completion, and as late if occurring thereafter [33].

### 2.2. Radiation and Hormonal Therapy

All treated patients underwent a non-contrast-enhanced simulation CT in the supine position, with 3 mm slice thickness, using a lower-limb immobilization system (Prostep^®^, Innovative Technologie Völp e.U., Innsbruck, Austria). Following internal protocol, each patient drank 500 mL of water, starting with an empty bladder, 20 min before the CT scan. For the therapeutic target contouring phase, the entire prostate gland and seminal vesicles were outlined in each slice as the clinical target volume (CTV), while the planning target volume (PTV) was generated by expanding the CTV with a margin of 5 mm posteriorly and 7 mm in all other directions, according to RTOG Intact Prostate Contouring Atlas [34]. MRI-CT fusion was utilized for target volume definition in the patients who underwent pre-treatment pelvic MRI. Organs at risk (OARs) included the bladder, rectum, bowel bag, penile bulb, and femoral heads.

Treatment was delivered via Volumetric Modulated Arc Therapy (VMAT) using four distinct fractionation regimens: moderate hypofractionation (2.5–2.7 Gy/fraction), extreme hypofractionation (6.1 Gy/fraction), or conventional fractionation (2.0 Gy/fraction). Planning goals ensured that at least 95% of the PTV received 95% of the prescribed dose. OAR sparing was achieved by strictly adhering to institutional dose–volume constraints, which were biologically adapted for each schedule (76 Gy/38 fr, 70 Gy/28 fr, 67.5 Gy/25 fr, and 42.7 Gy/7 fr) to maintain iso-effective protection of healthy tissues. A comprehensive summary of target delineation, dose prescriptions, and specific OAR constraints is provided in Table 1. The precision of treatment delivery was ensured by a rigorous Image-Guided Radiotherapy (IGRT) protocol, consisting of daily Cone-Beam CT (CBCT) verification before each session. This allowed for the compensation of inter-fraction variability and the use of reduced PTV margins, which was fundamental in sparing healthy tissues and minimizing gastrointestinal and genitourinary toxicities.

Androgen suppression was achieved via LH-RH analogs (Triptorelin, Leuprorelin, Goserelin), with simultaneous treatment with the antiandrogen Bicalutamide in the first two weeks to prevent “flare-up”, or LH-RH antagonists (Degarelix). ADT was administered before, during, and/or after RT, for a minimum of 6 months. In case of suspected adverse drug reaction, the medication would have been discontinued.

### 2.3. Endpoints and Statistical Analysis

The primary endpoints of the study were biochemical failure-free survival (BFFS) and disease-free survival (DFS), both measured from the date of initiation of the first oncological treatment (either ADT or RT). BFFS was defined as the interval to the first detection of biochemical recurrence, defined as a serum total PSA level ≥2 ng/mL above the post-radiotherapy nadir, according to the Phoenix criteria. DFS served as a composite endpoint, reflecting the time to the first occurrence of either biochemical failure or clinical-radiological detection of local recurrence or distant metastasis. Notably, the initiation of any salvage therapy in this cohort was strictly tied to the events captured within the DFS definition. Secondary objectives included metastases-free survival (MFS), prostate cancer-specific survival (PCSS), and OS, all calculated from the same unified time-zero. Specifically, MFS was defined as the time to the first detection of distant metastasis, while PCSS and OS were defined as the time to death from the disease and death from any cause, respectively. Acute and late toxicities were summarized using frequencies and percentages, categorized by grade according to the study cohort. For continuous variables, median values and ranges were calculated; survival distributions were estimated using the Kaplan–Meier method. Statistical analysis was performed using R software (version 4.5.2; R Foundation for Statistical Computing, Vienna, Austria).

## 3. Results

### 3.1. Patients and Treatment Characteristics

Forty-three patients meeting the inclusion criteria were included in the analysis. The median age at diagnosis was 76 years (range: 65–82). Baseline patient characteristics and comorbidities are summarized in Table 2. Notably, 88.4% of patients presented at least two cardiovascular risk factors, with approximately 50% having three or more. Preexisting conditions included heart disease (25.6%), diabetes (23.3%), and respiratory pathology (18.6%).

Regarding clinical and instrumental staging, all patients underwent physical examination and serum PSA measurement; 8 had a transrectal ultrasound (18.6%), 36 had a pelvic MRI (83.7%). Of the 43 total patients, 24 (55.8%) were staged using conventional imaging (CT and bone scan); the remaining 19 (44.2%) underwent PET/CT staging, utilizing either 18F-fluorocholine (N = 13) or 68Ga-PSMA-11 (N = 6) radiotracers.

The clinical T-stage was T4 in 1 patient (2.3%), T3a in 7 (16.3%), and T3b in 11 (25.6%); the remaining 24 patients (55.8%) were classified as cT2, with none classified as cT1.

The median pre-treatment PSA was 8.30 ng/mL (range: 1.30–107), with 8 patients (18.6%) exceeding 20 ng/mL. Histological analysis via biopsy revealed an ISUP Grade Group of 5 in 8 patients (18.6%) and 4 in 27 patients (62.8%); among these, 5 patients (11.6%) had a primary Gleason pattern 5; in 8 patients, the ISUP Grade Group was lower or equal to 3. According to the NCCN Risk Group classification, 41 patients (95.3%) were categorized as HR and 2 (4.7%) as very-HR. Notably, despite the relatively low median PSA and the prevalence of cT2 stages, 36 patients (83.7%) presented with ≥2 HR factors, confirming an aggressive disease profile for most of the cohort. The estimated risk of microscopic nodal involvement using the Roach equation was >15% in 38 patients (88%), and >7% in 40 patients (95%) using the MSKCC nomogram.

ADT was administered for a median duration of 24 months (range: 6–63 months). While most of the cohort received long-term ADT consistent with international guidelines for HR disease, 3 patients (6.9%) underwent a shortened course (6 months) due to low treatment compliance or the presence of severe baseline comorbidities.

Regarding the radiotherapy schedules, 36 patients (83.7%) received moderate hypofractionation, while extreme and conventional fractionation were utilized in 3 and 4 patients, respectively. The Dose Volume Histograms (DVH) are summarized in Table 3. Dosimetric analysis of the PTV showed excellent coverage across all fractionation schedules. The median V95% was 100% (range: 97–100%) for both the 42.7 Gy/7 fr and 67.5 Gy/25 fr protocols, and 98% (range: 97–98%) for the 70 Gy/28 fr protocol. For the rectum and bladder median values for both organs were consistently lower than the established tolerance thresholds. The rectal sparing was particularly efficient in the 70 Gy/28 fr and 42.7 Gy/7 fr cohorts. In the moderate hypofractionation (42.7 Gy) group, the median V27.47 was 18% (limit < 35%) and the V37.5 (absolute volume) was 8 cc, well below the 20 cc constraint. For the 67.5 Gy/25 fr group, although a wider range was observed (up to 41% for V43.86), the median remained at 26.5%, safely below the 35–45% threshold. The 70 Gy/28 fr schedule achieved the lowest rectal doses at high-dose levels, with a median V59.09 of only 4% (limit 15–20%). Bladder dosimetric parameters showed significant sparing throughout all treatment arms. For the 42.7 Gy protocol, the median V15 was 19%, significantly lower than the 50% limit. In the 67.5 Gy and 70 Gy groups, the median V52.63 and V54.55 were 11% and 13%, respectively, both well below the 35% constraint.

### 3.2. Clinical Outcomes and Toxicities

The median follow-up duration was 60 months (range: 10–96). Low PSA nadir values (median nadir: 0.01 ng/mL; range: 0.00–0.35 ng/mL) were observed across the entire cohort. According to Phoenix criteria, no patient experienced biochemical relapse, resulting in a 100% BFFS rate. However, four patients (9.3%) experienced radiological recurrence detected via 68Ga-PSMA PET/CT; these included two cases of bone metastases, two cases of loco-regional nodal recurrence and once case of isolated intraprostatic relapse (Table 4). Among the metastatic patients, one developed bone lesions 16 months after the initiation of oncological treatment (PSA 1.02 ng/mL), while the second was diagnosed at 75 months (PSA 1.44 ng/mL). Regarding nodal recurrences, the first patient was identified at 46 months (PSA 0.49 ng/mL, rising from a nadir of 0.21 ng/mL), and the second at 75 months (PSA 1.44 ng/mL).

**Figure 1:**
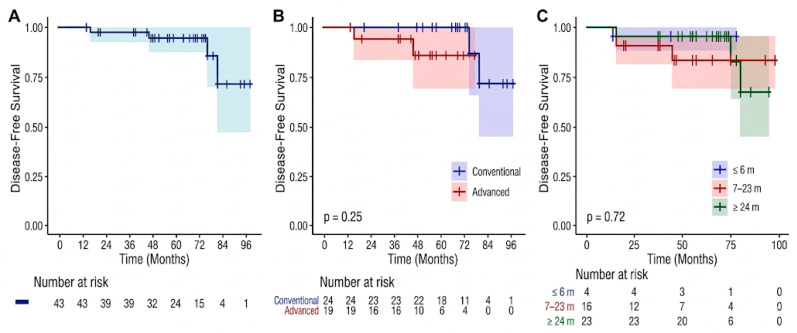
Kaplan–Meier estimates of Disease-Free Survival (DFS). (**A**) Overall DFS for the entire study population (n = 43), reflecting the time to any disease recurrence (local, regional, or distant). (**B**) DFS comparison between patients staged with conventional imaging (blue line) and those staged with advanced PET/CT imaging (red line). No statistically significant difference was reached (*p* = 0.25). (**C**) DFS stratified by the duration of Androgen Deprivation Therapy (ADT): ≤6 months (blue), 7–23 months (red), and ≥24 months (green), showing no significant impact of hormonal therapy duration on recurrence-free outcomes (*p* = 0.72). Shaded areas indicate 95% Confidence Intervals (CI). The number of patients at risk is provided below the *x*-axis.

**Figure 2:**
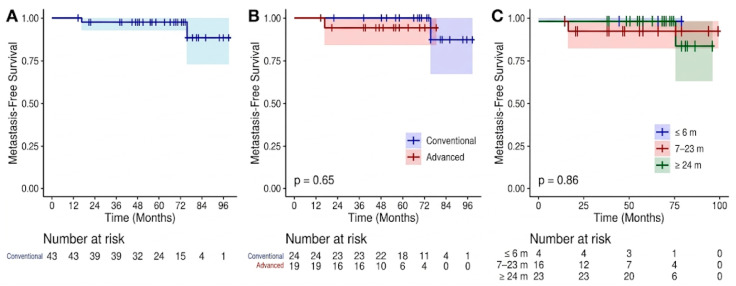
Kaplan–Meier estimates of Metastasis-Free Survival (MFS). (**A**) Overall MFS for the entire study cohort (n = 43), demonstrating high rates of stability over a median follow-up of 60 months. (**B**) MFS stratified by initial staging modality: conventional imaging (blue line) vs. advanced PET/CT imaging (red line). No statistically significant difference was observed between the two groups (*p* = 0.65). (**C**) MFS according to the duration of Androgen Deprivation Therapy (ADT): ≤6 months (blue), 7–23 months (red), and ≥24 months (green). The duration of hormonal therapy did not significantly impact MFS outcomes (*p* = 0.86). Shaded areas represent 95% Confidence Intervals (CI). The number of patients at risk is reported below each temporal milestone.

**Figure 3:**
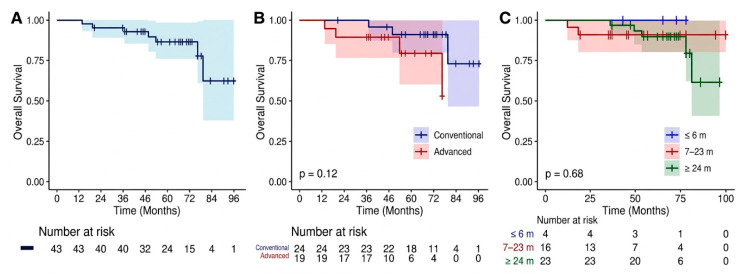
Kaplan–Meier estimates of Overall Survival (OS). (**A**) Overall survival for the entire cohort (n = 43) over the follow-up period. (**B**) OS stratified by initial staging modality: conventional imaging (blue line) vs. advanced PET/CT imaging (red line). No statistically significant difference in survival was observed between the two groups (*p* = 0.12). (**C**) OS according to the duration of Androgen Deprivation Therapy (ADT): ≤6 months (blue), 7–23 months (red), and ≥24 months (green). The duration of hormonal treatment did not significantly correlate with survival outcomes (*p* = 0.68). Shaded areas represent 95% Confidence Intervals (CI). The number of patients at risk is indicated below the *x*-axis.

Using the Kaplan–Meier method, the estimated 5- and 7-year DFS rates were 94.7% and 71.8%, respectively (Figure 1A), while the 5- and 7-year MFS rates were 97.6% and 88.7% (Figure 2A). Stratification by staging modality showed that the use of advanced imaging did not significantly impact these outcomes. Specifically, no statistically significant differences were observed between patients staged with 68 Ga-PSMA PET/CT and those staged with conventional imaging for either MFS (*p* = 0.65; Figure 2B) or DFS (*p* = 0.25; Figure 1B). During the follow-up period, 7 deaths occurred; however, none were attributable to PC progression. All deceased patients had at least two cardiovascular risk factors, with three also suffering from renal insufficiency and two from chronic obstructive pulmonary disease. Consequently, while the OS at 5 and 7 years was 86.7% and 62.4%, respectively (Figure 3A), the PCSS remained 100%. As with the other endpoints, the choice of initial staging modality did not significantly influence OS (*p* = 0.12; Figure 3B; Table 5).

RT-related toxicities are summarized in Table 6. Regarding acute genitourinary (GU) toxicity, 7 patients (16%) developed Grade 2 (G2) events, with no cases of G3. A single Grade 4 genitourinary (GU) toxicity (2.3%) was observed in a patient who experienced acute urinary retention (AUR) requiring emergency bladder catheterization and specialized urological management. The complication was successfully resolved within 30 days, with the subsequent removal of the catheter and restoration of normal voiding function. Acute gastrointestinal (GI) toxicity was low, with only 3 patients (7%) experiencing G2 symptoms and no cases of G3–G4. At late follow-up, 7 patients (16%) reported persistent G2 urinary discomfort. Late GI side effects were remarkably rare, except for one patient who developed G3 proctitis.

Other clinical issues reported during the follow-up, mostly while hormonal therapy was ongoing, included mild urinary incontinence (16%), chronic fatigue (14%), and sexual dysfunction (11%).

## 4. Discussion

This retrospective analysis reports our institutional experience in the curative treatment of elderly patients with HR-PC, without clinical evidence of lymph node involvement or distant metastases at baseline. All patients received PORT, omitting ENI, in combination with long-term ADT. Our strategy aligns with NCCN and EAU guidelines, which recommend 18 to 36 months of androgen suppression for HR disease [6,25]. The median ADT duration of 24 months in our cohort reflects adherence to these standards, even within an elderly population (median age 76). While recent trials explore intensification with second-generation anti-androgens, the high prevalence of cardiovascular comorbidities in our sample (88% with ≥2 risk factors) justified a more conservative approach. We focused on standard ADT to minimize metabolic and cardiac toxicity without compromising biochemical control.

Clinical outcomes demonstrated an excellent response to this strategy. Distant bone metastasis occurred in only two cases, and nodal recurrence in only one, consistent with the existing literature [30]. Notably, in patients who experienced recurrence, serum PSA levels did not reach the biochemical recurrence (BCR) threshold defined by the Phoenix criteria (nadir + 2 ng/mL). However, a progressive rise in PSA (exceeding 1 ng/mL) was associated with concomitant radiological progression. Consequently, salvage therapy was initiated before the formal Phoenix threshold was met. This suggests that in the era of advanced imaging, such as PSMA PET/CT, radiological findings may anticipate biochemical relapse. This clinical “failure” prior to the formal nadir + 2 definition indicates that the Phoenix criteria might require updating to better reflect current clinical practice and the higher sensitivity of modern diagnostics. Regarding survival, the seven deaths recorded during follow-up were attributed to significant pre-existing comorbidities; none were cancer-specific. This underscores the impact of competing mortality, a hallmark of geriatric oncology. In our cohort, the risk of death from non-oncological causes often outweighed the risk of PC-specific mortality. While the sample size precluded a formal competing-risk model, the median follow-up of 60 months is clinically sufficient to assess both treatment safety and medium-term oncological control. In this specific population, short-to-medium-term outcomes often hold greater clinical relevance than long-term estimates, as treatment decisions must meticulously balance oncological benefit against life expectancy and quality of life.

To date, few prospective randomized Phase III trials have compared PORT with ENI in clinically N0 HR patients, and their findings remain largely conflicting. Notably, none of these studies observed a significant difference in OS. Furthermore, these trials did not exclusively enroll patients currently classified as HR or very-HR, nor did they specifically target the elderly population, which represents a distinct clinical challenge. The GETUG-01 study, reporting long-term results after a median follow-up of 11.4 years, enrolled patients with heterogeneous prognostic characteristics [20]. The HR subgroup in that study included cases that would today be classified as intermediate-risk. Among the 354 HR patients, no significant 10-year difference was found between pelvic irradiation and PORT regarding Event-Free Survival (EFS) (52% vs. 54.2%), which encompassed both biochemical recurrence and clinical progression. In GETUG-01, RT was delivered using either conventional or moderate hypofractionation, but ADT was limited to only 4–8 months, and the median age was 70 years—significantly younger than our cohort. Similarly, the NRG/RTOG 9413 multicenter trial employed a 2 × 2 factorial design to evaluate pelvic irradiation and ADT timing [35]. While significant differences were noted in 10-year Progression-Free Survival (PFS) (*p* = 0.002), these were heavily influenced by the sequence of ADT and RT rather than the nodal irradiation alone. Although neoadjuvant ADT followed by ENI showed some benefit, no significant differences were observed in loco-regional recurrence or MFS. Crucially, ENI was associated with a trend toward increased acute and late gastrointestinal toxicity. Several limitations in these landmark trials must be highlighted when considering current clinical standards. First, both studies predominantly utilized conventional fractionation at what are now considered suboptimal doses (70 Gy). Second, the duration of ADT was notably short (4–8 months). In contrast, current international guidelines recommend long-term androgen deprivation (18–36 months) for HR disease.

Recent evidence from the POP-RT phase III trial (2021) showed a significant 5-year advantage for Whole-Pelvis Radiotherapy (WPRT) over PORT in terms of BFFS (95% vs. 81.2%, *p* < 0.0001) and Metastasis-Free Survival MFS [23]. Interestingly, while POP-RT used—similarly to our study—the Phoenix criteria, our cohort achieved a 100% BFFS rate. This discrepancy is likely driven by our older population and systematic long-term ADT, which may further suppress PSA kinetics. Notably, the POP-RT cohort had a lower median age (66 years), and subgroup analysis revealed that the benefit of pelvic irradiation was significantly more pronounced in younger patients (*p* = 0.03). Furthermore, 80% of POP-RT patients were staged with 68Ga-PSMA PET/CT, a highly sensitive modality that may have identified a subset of patients with low-volume nodal disease who are more likely to benefit from ENI.

This age-dependent benefit was further corroborated by Sayan et al. (2024) [36]. In their post-randomization analysis, WPRT was associated with a 10-year reduction in All-Cause Mortality (ACM) only in men younger than 65 years (8.42% vs. 30.10%), whereas no benefit was observed in those aged 65 or older (28.51% vs. 28.70%). Similarly, the retrospective analysis by Amini et al. on over 14,000 HR patients found no OS advantage for WPRT [37].

These findings, coupled with the PIVOTAL trial’s report of significantly higher Grade ≥2 gastrointestinal toxicity in the pelvic arm (26% vs. 7%) [38], support a more tailored approach for the elderly. As the International Society of Geriatric Oncology (SIOG) emphasizes, curative-intent treatment in patients over 70 must carefully balance oncological goals against life expectancy and the presence of irreversible comorbidities [39].

Our study has several limitations that warrant consideration. First, the sample size is relatively small, although it represents a highly selective cohort meeting strict risk and age criteria. Second, while most patients received long-term ADT, there was some heterogeneity in ADT duration.

A further limitation is the transition in staging pathways from conventional imaging (CT and bone scan) to PET/CT during the 2016–2022 period. Given that PSMA-PET/CT offers significantly higher accuracy (92%) than conventional imaging (38%) for detecting pelvic nodal metastases, an understaging bias in the earlier cohort cannot be excluded. This shift reflects the “real-world” evolution of clinical practice but necessitates caution in interpreting longitudinal results.

Finally, the lack of data on testosterone recovery is a notable limitation. In elderly patients, persistent castration following ADT is common and may influence PSA kinetics. We hypothesize that this potential synergy between prolonged ADT and radiotherapy could reinforce the rationale for de-escalating radiation volumes. By omitting ENI in patients with potentially sustained biochemical responses, treatment-related toxicities can be minimized without necessarily compromising oncological safety. This hypothesis deserves further investigation in prospective geriatric oncology trials.

## 5. Conclusions

In summary, our findings support the use of PORT combined with long-term ADT as a safe and effective strategy for elderly patients with HR-PC. While ENI remains controversial, our data suggest that a de-escalated approach does not compromise mid-term oncological control. In a geriatric population characterized by significant comorbidity, prioritizing the reduction in treatment-related toxicity through volume de-intensification aligns with the principles of personalized medicine, balancing clinical efficacy with the maintenance of quality of life.

## Figures and Tables

**Table 1:** Target volume and OARs delineation and their dose constraints.

Target Volume Delineation and Dose Prescription				
CTV Prostate	Entire prostate gland and seminal vesicles.			
PTV Prostate	Expansion of 7 mm around the CTV prostate in all directions except posteriorly (5 mm).			
OAR Delineation and Dose Constraints				
		Dose prescription (Gy)/Fractions (N)		
	42.7/7 fr	67.5/25 fr	70/28 fr	76/38 fr
Rectum	Solid structure starting from recto sigmoid flexure up to the bottom of ischial tuberosity.			
	V(27.47) < 35%	V(43.86) < 35–45%	V(45.45) < 35–45%	V(50) < 35–45%
	V(32.97) < 25%	V(52.63) < 25–30%	V(54.55) < 25–30%	V(60) < 25–30%
	V(35.71) < 15%	V(57.02) < 15–20%	V(59.09) < 15–20%	V(65) < 15–20%
	V(37.50) < 20 cc	V(61.40) < 1–3%	V(63.64) < 1–3%	V(70) < 1–3%
Bladder	Solid structure from the dome to the base including the wall.			
	V(15) < 50%	V(52.63) < 35%	V(54.55) < 35%	V(60) < 35%
	V(18.10) < 40%	V(61.40) < 20%	V(63.64) < 20%	V(70) < 20%
	V(32.97) < 35%			
	V(38.46) < 20%			
Penil Bulb	Bulbous portion of the corpus spongiosum located inferior to the urogenital diaphragm.			
	Dmean < 25 Gy	Dmean < 39.47–43.86 Gy	Dmean < 40.91–45.45 Gy	Dmean < 45–50 Gy
Femur Heads	Drawn within the acetabulum without including the neck of femur.			
	V(21.98) < 2%	V(35.09) < 2–4%	V(36.36) < 2–4%	V(40) < 2–4%
	Dmax < 24.73 Gy	Dmax < 39.47 Gy	Dmax < 40.91 Gy	Dmax < 45 Gy
Bowel Bag	Single solid structure encompassing the peritoneal cavity and any pelvic bowel loops.			
	V(18.10) < 5 cc	Dmax < 46.81 Gy	Dmax < 48.89 Gy	Dmax < 55 Gy
	V(30) < 1 cc			
	V(35) < 0.5 cc			
	Dmax ≤ 27.50 Gy			

Abbreviations: CTV, Clinical Target Volume; PTV, Planning Target Volume; OAR, Organs at Risk; V(D), percentage of the organ volume receiving at least the dose D (in Gray); V(D) < Xcc, absolute volume of the organ in cubic centimeters receiving at least the dose D; Dmean, mean dose; Dmax, maximum dose; Gy, Gray; cc, cubic centimeters; N, number of fractions.

**Table 2:** Baseline clinical and treatment characteristics.

Characteristic		N of Events (%)
Median Age (years)		76 (65–82)
Median PSA (ng/mL)		8.30 (1.30–107)
PSA > 20 (ng/mL)		8 (18.6)
PSA < 20 (ng/mL)		35 (81.4)
Gleason Score	6	2 (4.7)
	7 (3 + 4)	1 (2.3)
	7 (4 + 3)	5 (11.6)
	8	27 (62.8)
	9	6 (14.0)
	10	2 (4.7)
ISUP	1	2 (4.7)
	2	2 (4.7)
	3	4 (9.3)
	4	27 (62.8)
	5	8 (18.6)
Tumor Stage	T1	0 (0)
	T2	24 (55.8)
	T3a	7 (16.3)
	T3b	11 (25.6)
	T4	1 (2.3)
Androgen Deprivation Therapy	LHRH analogs	35 (81.4)
	LHRH antagonist	8 (18.6)
	Only neoadjuvant	1 (2.3)
	Neoadjuvant, concurrent, adjuvant	37 (86.0)
	Concurrent and adjuvant	2 (4.7)
	Only adjuvant	3 (7.0)
	≤6 months	4 (9.3)
	>6 < 24 months	16 (37.2)
	≥24 months	23 (53.5)
Baseline imaging	TRUS	8 (18.6)
	MRI	36 (83.7)
	CT	34 (79.1)
	Bone Scan	37 (86.0)
	68Ga-PSMA-11 PET	6 (14.0)
	18F-fluorocholine PET	13 (30.2)
Comorbidities	≥2 Cardio-vascular risk factors	38 (88.4)
	≥3 Cardio-vascular risk factors	22 (51.2)
	Cardiac disease	11 (25.6)
	COPD, pulmonary emphysema	8 (18.6)
	Gastro-intestinal disease	24 (55.8)
	IRC, kidney transplant	4 (9.3)
	Diabetes	10 (23.3)
TURP		10 (23.3)
RT total dose/fractions	76/38	1 (2.3)
	70/28	3 (7.0)
	67.5/25	36 (83.7)
	42.7/7	3 (7.0)
LNI Risk	Roach Formula Median	(27)
	Roach Formula > 15%	38 (88.4)
	MSKCC Nomogram Median	(36)
	MSKCC Nomogram > 7%	40 (93.0)
Risk group (NCCN 2026 [25])	Very Low	0 (0)
	Low	0 (0)
	Intermediate	0 (0)
	High	41 (95.3)
	Very High	2 (4.7)

Abbreviations: PSA, prostate-specific antigen; ISUP, International Society of Urological Pathology; LHRH, luteinizing hormone-releasing hormone; TRUS, transrectal ultrasound; MRI, magnetic resonance imaging; CT, computed tomography; 68Ga-PSMA-11 PET, Gallium-68 prostate-specific membrane antigen positron emission tomography; 18F-fluorocholine PET, Fluorine-18-fluorocholine positron emission tomography; COPD, chronic obstructive pulmonary disease; TURP, transurethral resection of the prostate; RT, radiotherapy; LNI, lymph node involvement; MSKCC, Memorial Sloan Kettering Cancer Center; NCCN, National Comprehensive Cancer Network.

**Table 3:** Summary of dose–volume histogram (DVH) parameters for OARs and PTV coverage across different fractionation schedules.

DVH Summary						
	Dose Prescription (Gy)/Fractions (N)					
	42.7/7 fr	Median (Range)	67.5/25 fr	Median (Range)	70/28 fr	Median (Range)
Rectum	V(27.47)	18% (17–18)	V(43.86)	26.5% (6–41)	V(45.45)	12% (12–13)
	V(32.97)	12% (11–13)	V(52.63)	17.5% (4–32)	V(54.55)	7% (6–7)
	V(35.71)	9% (8–10)	V(57.02)	13.5% (3–26)	V(59.09)	4% (3–5)
	V(37.50)	8 cc (7–8)			V(63.64)	3% (2–3)
Bladder	V(15)	19% (18–38)	V(52.63)	11% (4–76)	V(54.55)	13% (10–18)
	V(18.10)	17% (17–36)	V(61.40)	8% (3–68)	V(63.64)	10% (7–11)
	V(32.97)	8% (7–21)				
	V(38.46)	5% (5–15)				
PTV Dose95 Median (range)	100% (97–100)		100% (97–100)		98% (97–98)	

Abbreviations: DVH, dose–volume histogram; OARs, organs at risk; PTV, planning target volume; Gy, Gray; N, number of fractions; V(x), volume of the organ receiving at least x Gy; Dose95 (%), percentage of the prescribed dose covering 95% of the planning target volume.

**Table 4:** Summary of clinical recurrence and mortality events during follow-up. The upper section details patients with radiological progression, while the lower section lists mortality events. Notably, all deaths were attributed to non-prostate cancer-related causes.

ID	Event	Time to Event (Months)	PSA at Event (ng/mL)	Imaging
8	Bone and lymph nodes metastasis	75	1.44	18F-fluorocholine PET
40	Prostate and lymph nodes	47	0.49	68Ga-PSMA-11 PET
43	Bone metastasis	40	1.02	68Ga-PSMA-11 PET
4	Death	78	0.02	
5	Death	55	0.04	
7	Death	38	0.01	
10	Death	82	0.00	
17	Death	53	0.18	
20	Death	15	0.01	
42	Death	21	0.01	

Abbreviations: PSA, prostate-specific antigen; 68Ga-PSMA-11, Gallium-68 prostate-specific membrane antigen; 18F-fluorocholine, Fluorine-18-fluorocholine.

**Table 5:** Summary of Survival Outcomes (n = 43). Kaplan–Meier estimates for Biochemical Failure-Free Survival (BFFS), Disease-Free Survival (DFS), Metastasis-Free Survival (MFS), Prostate Cancer-Specific Survival (PCSS), and Overall Survival (OS) at 5-year and 7-year intervals.

Outcome	Events	KM 5-Years	KM 7-Years
BFFS	0	100%	100%
DFS	4	94.7%	71.8%
MFS	2	97.6%	88.7%
PCSS	0	100%	100%
OS	7	86.7%	62.4%

Abbreviations: BFFS, biochemical failure-free survival; DFS, disease-free survival; MFS, metastasis-free survival; PCSS, prostate cancer-specific survival; OS, overall survival; KM, Kaplan–Meier method; Events, number of recorded clinical or mortality events.

**Table 6:** Acute and late toxicities.

Acute Toxicities		
	Grade	N° of Events (%)
Genitourinary	0	10 (23.3)
	I	25 (58.1)
	II	7 (16.3)
	III	0 (0)
	IV	1 (2.3)
Gastrointestinal	0	21 (48.8)
	I	19 (44.2)
	II	3 (7.0)
	III	0 (0)
	IV	0 (0)
Late toxicities		
	Grade	N° of events (%)
Genitourinary	0	25 (58.1)
	I	11 (25.6)
	II	7 (16.3)
	III	0 (0)
	IV	0 (0)
Gastrointestinal	0	36 (83.7)
	I	6 (14.0)
	II	0 (0)
	III	1 (2.3)
	IV	0 (0)

## Data Availability

The data presented in this study are available on request from the corresponding author.
